# Photocatalytic Bacterial Destruction and Mineralization by TiO_2_-Based Photocatalysts: A Mini Review

**DOI:** 10.3390/molecules29102221

**Published:** 2024-05-09

**Authors:** Paulina Rokicka-Konieczna, Antoni W. Morawski

**Affiliations:** Department of Inorganic Chemical Technology and Environment Engineering, Faculty of Chemical Technology and Engineering, West Pomeranian University of Technology in Szczecin, Pułaskiego 10, 70-322 Szczecin, Poland; antoni.morawski@zut.edu.pl

**Keywords:** TiO_2_, photocatalysis, photocatalytic bacterial mineralization, photocatalytic bacterial destruction

## Abstract

This work presents an overview of the reports on the bacterial cell photocatalytic destruction and mineralization process in the presence of TiO_2_-based photocatalysts. The presented research included experiments conducted in air and water. Numerous works confirmed that a photocatalytic process with TiO_2_ led to bacteria and their organic residues’ mineralization. Additionally, based on the obtained results, a possible two-stage mechanism of photocatalytic mineralization in the presence of TiO_2_-based materials was proposed. To help future studies, challenges of photocatalytic microorganism mineralization are also proposed. There are some aspects that need to be addressed, such as the lack of standardization of conducted research or relatively small amount of research on photocatalytic microorganism mineralization. According to our best knowledge, in the available literature, no work regarding a summary of previous research on photocatalytic bacterial mineralization process was found.

## 1. Introduction

Increased awareness of environmental protection results in increased interest about new sustainable methods of dealing with harmful pollutants. New technologies of water and air purification should face a number of requirements, such as efficiency, low cost, and minimum environmental impact. It seems that the method of photocatalytic oxidation with titanium dioxide (TiO_2_) fulfills the above-mentioned conditions.

Photocatalysis has come to global attention as a “green” advanced oxidation process that can be used in various applications, such as water treatment, air purification, or self-cleaning glass coating [1,2]. Among many metal oxide semiconductors, titanium dioxide is considered as one of the most popular and significant photocatalyst. Its great popularity is due to such properties as chemical stability, non-toxicity, and relatively inexpensive [3,4].

TiO_2_-based photocatalysts, due to their properties, have been widely used in a variety of applications and products, including environmental and energy-related fields. Among the most important are self-cleaning surfaces [5], carbon dioxide (CO_2_) conversion to chemical fuels [6], water reduction to produce hydrogen [7] for the decomposition of organic pollutants [8], and chemical degradation, e.g., for environmental remediation [9,10].

TiO_2_-based materials have also been extensively examined because of their ability to inactivate pathogenic microorganisms (such as bacteria, viruses, fungi, algae). Therefore, TiO_2_ photocatalysis has been recognized as an effective disinfection technique in wastewater treatment, food protection applications, biomedical applications, or disinfection. In the available literature, there are many summary papers concerning the application of TiO_2_ materials in antibacterial and environmental fields. Jafari and co-workers [11] reviewed the biomedical application of TiO_2_ nanomaterials. The review provides information about TiO_2_-based nanomaterial applications in drug delivery, medical implants, and antibacterial fields. Kodithuwakku et al. [12] have reviewed recent developments in the structural modification of TiO_2_, which are applied in food packaging applications. The use of TiO_2_ photocatalysts in microorganism inactivation and disinfection was discussed by Reddy et al. [13], Demirel at al. [14], Amiri et al. [15], Bono et al. [16], and Pasquale et al. [17]. Bono and co-workers [16] have made a literature review concerning the effect of TiO_2_-photocatalysis on airborne bacteria and viruses. Pasquale et al. [17] have additionally analyzed the use of TiO_2_-based photocatalytic nanomaterials in surface disinfection. The mentioned works are just some of the available literature on the subject.

One of the most important advantages of photocatalysis is the degradation of impurities into less toxic end compounds or even their total mineralization to CO_2_ and H_2_O [18]. The great majority of studies refers to the photocatalytic mineralization of organic pollutants and antibiotics [19,20,21]. The effectiveness of TiO_2_ photocatalysis on the bacterial mineralization process in air and aquatic environments has also been examined by several researchers. What is more, it has been shown that the total mineralization of bacterial cells is possible [18,22]. However, the number of papers on photocatalytic bacterial mineralization is relatively small compared to the total number of papers on photocatalytic bacterial inactivation or photocatalytic water disinfection. It should be mentioned that the first reference to photocatalytic microorganism mineralization dates back to 1998 [18]. Since then, this topic has appeared from time to time in the literature, but there are still many questions and issues to be explored. Additionally, no work regarding a summary of previous research in the literature was found. The mechanism of photocatalytic bacterial mineralization has also not been well studied and summarized.

This paper aims to provide a brief overview of articles concerning photocatalytic bacterial mineralization. First, the mechanisms of photocatalytic bacterial inactivation were briefly described because there is no bacterial mineralization without microorganism inactivation. Second, the research concerning photocatalytic bacterial mineralization are presented. This review also includes reports concerning photocatalytic bacterial destruction, photocatalytic bacterial cell decomposition, or photocatalytic bacterial oxidation, in which the authors suggested the possible mineralization of bacteria or their organic material on the TiO_2_ surface during the photocatalytic process. Additionally, emphasis was placed on such information as the type of photocatalyst, test methods used to determine the mineralization process, used microorganism, and time of experiment. Based on previous research in this field, an attempt to describe the mechanism of the photocatalytic mineralization of bacteria will also be undertaken. Finally, challenges and perspectives in future research on photocatalytic microorganism mineralization are presented.

## 2. The Mechanisms of Photocatalytic Bacterial Inactivation—Fundamentals

In the literature concerning the subject of the antimicrobial activity of TiO_2_-based photocatalysts and TiO_2_-based nanomaterials, different terms and research are applied. The most common research involves the photocatalytic disinfection of microorganisms—a process during which they are inactivated. According to the literature, the term “bacterial inactivation” refers to the reduction or complete termination of bacterial growth, mainly due to damage in DNA or protein synthesis. This process is often accompanied by impairment of cellular integrity [23]. However, in most cases dead bacterial cells are presented and identifiable. The term “bacterial inactivation” is often used interchangeably with “bacterial destruction”. In this case, beyond a loss of viability, obvious damage of the bacterial cell (damage to the peptidoglycan layer, distortion of treated cells) can be observed [24]. Sometimes, only residuals or parts of bacterial cells are observed. In turn, “photocatalytic mineralization” means the complete decomposition of organic substances into simple solid inorganic compounds (such as CO_2_ and H_2_O).

Considering the process of photocatalytic bacterial mineralization, first, the mechanism of photocatalytic bacterial inactivation should be briefly mentioned. The discovery that gave rise to research on the antimicrobial properties of titanium dioxide was the removal of *Escherichia coli* and *Lactobacillus acidophilus* bacteria and *Saccharomyces cerevisiae* yeast from water using a photoelectrochemical process using a Pt/TiO_2_ electrode. The experiment was performed by a research team under the direction of Professor Matsunaga [25]. Additionally, authors observed that the photocatalytic process caused the photooxidation of Coenzyme A (CoA), which in turn led to the inhibition of cell respiration and finally cell death. Since then, research on photocatalytic microorganism inactivation has become increasingly popular, and year by year, the number of works on this topic increased. It has been shown that the photocatalytic process is effective against a wide range of microorganisms, such as bacteria, viruses, fungi, algae, and even cancer cells. In the available literature, there are many very good and interesting review papers on TiO_2_-based materials for antimicrobial application [12,13,14,15,16,17,24].

Although the antimicrobial properties of TiO_2_ have been confirmed many times, the biocidal mechanism has not been fully elucidated. It is believed that the antimicrobial activity of titanium dioxide is multifactorial and involves many mechanisms [26]. When analysing the mechanisms and models of photocatalytic bacterial inactivation, at the beginning, attention should be paid to understanding the interactions between the photocatalyst and microorganism cells. Many studies have found that the degree of bacterial cell damage is strongly related to the photocatalyst form—namely whether it is immobilized on a carrier or suspended in a reaction suspension. Numerous studies confirmed that titanium dioxide suspended in a solution presents higher biocidal activity than a photocatalyst in an immobilized form [24]. This may be the result of facilitated contact between bacterial cells and TiO_2_ particles, which can lead to physical damage of bacterial cells. Based on the photocatalyst crystallite size and the way it interacts with bacterial cells, Dalrymple et al. [26] proposed three types of photocatalyst–bacteria interactions. In the first one, the photocatalyst particles are bigger than the bacterial cells, e.g., as a result of the agglomeration of the photocatalyst particles or when the photocatalysts takes the form of a continuous immobilized surface (such as in the case of thin film). Bacterial cells can adhere to a single particle of the photocatalyst or to its entire surface. In the second one, the photocatalyst particles present a much smaller size than the bacterial cells. This is the case when the photocatalyst (usually suspended in a solution) is characterized by very small crystallite sizes (about 10 nm—20 nm). In this case, the photocatalyst particles may enter the bacterial cell and initiate internal photocatalytic reactions inside them—which can significantly increase its biocidal activity. In the third mechanism, the photocatalyst particles and microbial cells are of a similar size. They remain in a heterogeneous system and interact with each other. In each of the above-mentioned models, bacterial cells may be physically damaged by particles of photocatalysts.

In view of that, very close or even direct contact of photocatalysts with the surface of the bacterial cell could be a significant factor in its biocidal activity; it is also necessary to mention electrostatic interactions between TiO_2_ and microorganisms in aquatic environments [27]. The surface charge can be evaluated based on zeta potential—which is the electrical potential between the bacterial surface and the surrounding aquatic environment. Bacterial cells are characterized by a negative electrostatic surface. In turn, the zeta potential value of TiO_2_ nanoparticles may be different depending on different titanium precursors, the method of TiO_2_ synthesis, the chemical composition of the particle surfaces, TiO_2_ modification, etc. [28,29]. The opposite surface charges of the bacteria (negative zeta potential) and TiO_2_-based photocatalysts (positive zeta potential) promotes their interactions and, therefore, may cause their faster inactivation.

**Figure 1:**
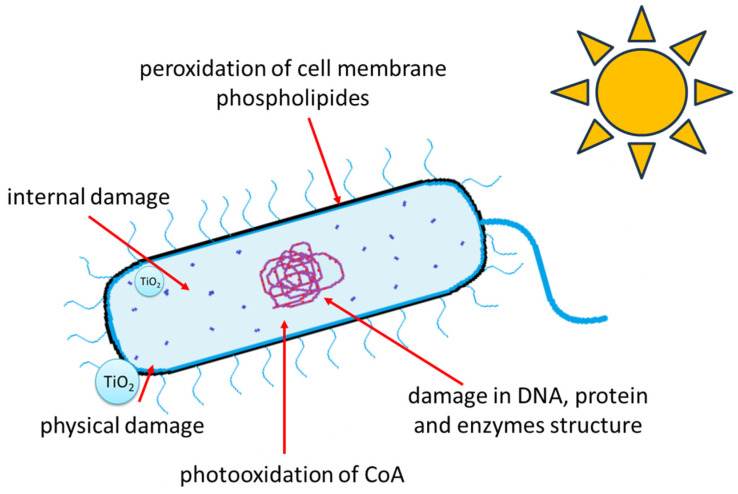
Summary of mechanisms leading to photocatalytic bacterial inactivation.

Regardless of the differences in size or electrostatic charge between the photocatalyst and the bacterial cells, the occurrence of reactive oxygen species (ROS) is considered the main factor leading to bacterial cell death. hydroxyl radicals, •OH (despite their short lifetimes), are characterized by the highest oxidation potential. Superoxide anion radicals (O_2_^•−^) are long-lived, but due to the negative charge, they cannot penetrate inside the bacterial cells (they are being repelled from them). Reactive oxygen species (generated during the photocatalytic process) lead to gradual damages of the peptidoglycan, which is part of the cell wall. Then, the oxidation of cell membrane components is observed. Various research teams assumed different criteria for assessing the damage that has occurred. Saito et al. [30], Hu et al. [31], and Gogniat et al. [32] studied the leakage of potassium ions (K^+^), which indicated the increase in the cell membrane permeability of *Streptococcus sobrinus*, *Staphylococcus aureus*, and *E. coli*, respectively. The leakage of K^+^ ions disrupted the integrity of the cell membrane and caused the disappearance of the functional membrane potential, which finally led to bacterial cell death. The products formed during the oxidation of compounds that constitute the membrane structures of bacteria were also examined. It was found, for example, that the most susceptible to oxidation are fatty acids (especially polyunsaturated), which are a part of phospholipids. Peroxidation (the process of fatty acid oxidation that can occur during the photocatalytic process) has been confirmed by Maness et al. [33] and by Kiwi and Nadtochenko [34]. The oxidation of unsaturated fatty acids could also lead to the formation of superoxide radicals, which further react with neighbouring lipid molecules, generating the formation of lipid radicals (peroxyl and alkoxyl). Figure 1 schematically illustrates the process of photocatalytic bacterial inactivation.

The generation of reactive oxygen species also induce the oxidative interference of proteins, metabolic activities, or damage in bacterial DNA and enzyme structures. Carré et al. [35] studied the damage of various proteins in *E. coli* during the photocatalytic process. The authors observed that the main targets of the free radical attack were proteins in the cell membrane and enzymes involved in protective responses to oxidative stress. Koizumi et al. [36], Kumar et al. [37], and Rokicka et al. [38] observed that TiO_2_ caused a decrease in the intracellular superoxide dismutase, glutathione, and catalase activity. This has led to a decrease in cellular antioxidant defense and ROS accumulation, which finally caused bacterial death. Zhang et al. [39] also observed that ROS produced during the photocatalytic process as a result of binding to catalase could cause the disorders in enzyme second and tertiary structure. Gogniat and Dukan [40] proposed that DNA damage may be caused by the process of hydroxyl radical formation in the Fenton reaction. Fe^2+^ ions, which are part of the enzymes active centers (e.g., superoxide dismutase SOD), react with hydrogen peroxide, which is formed on the TiO_2_ surface. Finally, they could be able to generate a certain amount of “extra” hydroxyl radicals in vivo or ex vivo reactions.

In turn, Liu et al. [41] categorized the disinfection mechanisms of different photocatalysts into three aspects. In addition to the two mentioned above (physical damage to the cells by TiO_2_ particles and the chemical oxidation of ROS), the toxicity of metal ions that may be released from metal-modified photocatalysts are also mentioned.

## 3. Photocatalytic Bacterial Mineralization

### 3.1. Photocatalytic Bacterial Mineralization in Air

According to some sources [42,43], the Goswami et al. reports [44,45] can be considered the pioneering work on complete bacterial destruction in air via the photocatalytic process. The authors examined the effectiveness of the photocatalytic process in the inactivation of *Serratia marcescens* in air in closed spaces. Research was conducted in UV-transparent air recirculation systems, covered by a thin nano-TiO_2_ layer. Total destruction of the bacterial cells was observed after 5 h of the photocatalytic process [44]. In the following work, the time needed for total bacterial destruction was reduced to less than 3 min [45]. Admittedly, the authors themselves did not literally use the term “mineralization”, but they did not observe dead bacterial cell masses and their residues on TiO_2_-coated air filters after photocatalytic process. This could indicate that the mineralization of the microorganism occurred.

One of the first works in which the term “mineralization” was literally used is a paper published by Jacoby et al. [18]. The authors examined *E. coli* mineralization in the air in the presence of P25-coated glass activated by UV light. In order to confirm bacterial mineralization, three methods (scanning electron microscopy (SEM), ^14^C radioisotope labelling, and measurements of evolved CO_2_) were applied. The SEM analysis showed that after 75 h of the photocatalytic process, no *E. coli* bacterial cell on TiO_2_-coated glass was observed. Products of complete bacterial cell mass mineralization were confirmed with ^14^C radioisotope labeling. A total of 51% of the radioactivity from the added *E. coli* cell mass was recovered as a product of complete bacterial cell mass mineralization. The CO_2_ measurements using gas chromatography also indicate a 54% mineralization of the bacterial cell by photocatalytic oxidation. As the authors themselves pointed out, the above-mentioned work provides the first evidence that the organic matter derived from a bacterial cell can be completely oxidized. In the following works, Wolfrum and Jacoby [46] examined the photocatalytic mineralization of *E. coli*, *Micrococcus luteus*, *Bacillus cereus* (both cells and spores), and *Aspergillus niger* spores on P25-coated quartz disks. This time, the authors also analysed the amount of evolved CO_2_, but the obtained results have been additionally based on kinetic data and carbon mass balance. In addition, the analysis of the results over the photocatalytic destruction of various microorganisms showed no significant difference in the oxidation rates between Gram-positive and Gram-negative bacteria and between bacteria and their spores. In turn, *A. niger* spores have proven more resistant to photocatalytic oxidation; therefore, the time needed for the total oxidation of their spores must be significantly longer.

Kiwi and Nadtochenko [47] and Nadtochenko et al. [48] examined the photocatalytic peroxidation of *E. coli* membrane wall components (lipopolysaccharide, phosphatidyl-ethanolcholine, and peptidoglycan) on TiO_2_ porous films using attenuated total reflectance–Fourier transform infrared (ATR-FTIR) spectroscopy. The authors have taken into account kinetics of the ATR-FTIR spectra of *E. coli* and membrane wall components (the integral absorbance of ATR-FTIR spectra as a function of time) during photocatalytic peroxidation. It was noted that changes in spectral bands suggest the mineralization of organic material on the TiO_2_ surface during the photocatalysis process. According to the observation, lipopolysaccharide was the easiest wall component to mineralize. In turn, the time needed for peptidoglycan mineralization was more than 10 times longer [47].

The photocatalytic mineralization of aerosol-deposited microorganisms was also examined by Kozlova et al. [22]. The mineralization of *Bacillus thuringiensis* on slides with TiO_2_ and Pt/TiO_2_ was performed. In this case, similarly as in previous works, the photocatalytic mineralization of microorganisms was studied based on the CO_2_ evolution measurements. Additionally, the impact of different photocatalyst and microorganism loadings on the course of the mineralization process was examined. The research has shown that the completeness of the mineralization process depended on the photocatalyst-to-bacteria mass ratio. The rate of the photocatalytic CO_2_ production (from decomposed bacterial cells) increased with both the cell mass increase and the photocatalyst mass increase.

The summary of the studies concerning the photocatalytic bacterial mineralization in air was presented in Table 1.

### 3.2. Photocatalytic Bacterial Mineralization in Aqueous Solutions

Sökmen et al. [50] was one of the first who undertook research on photocatalytic bacterial mineralization in water suspension. The authors have shown a complete mineralization of *E. coli* in aqueous solutions containing TiO_2_ and silver-loaded TiO_2_. In the presented work, the formation of malondialdehyde (MDA), which is a lipidperoxidation product formed during the oxidation of bacterial membrane phospholipids (e.g., phosphatidylethanolamine), was monitored. First, the MDA formation during the photocatalytic degradation of *E. coli* on TiO_2_ and Ag-TiO_2_ neat was measured. Then, the photocatalytic degradation of MDA was examined. It was observed that all *E. coli* bacteria was degraded to MDA via the lipidperoxidation process. MDA in turn was further degraded to simpler compounds and finally to harmless products, such as CO_2_ (which was confirmed by the gas chromatography analysis).

The photocatalytic mineralization of *E. coli* in the membrane photocatalytic oxidation (MPCO) reactor was presented by Sun et al. [51]. The authors observed bacterial mineralization in the presence of the TiO_2_–Fe_2_O_3_ composite. The impact of such parameters as dissolved oxygen, the time of hydraulic retention, and bacterial concentration on removal process efficiency were examined. Experimental results revealed that the reaction rate of the photomineralization process could be described by pseudo-first-order kinetic behaviour by the role of dissolved molecular oxygen.

Photocatalytic bacterial mineralization was also confirmed in the presence of a composite of TiO_2_ nanotubes/Ti plates modified by g-C_3_N_4_ and SnO_2_ [52]. In this case, complete *E. coli* mineralization was observed after 32 h of the photocatalytic process. In turn, Cheng et al. [53], who examined the photocatalytic oxidation of *Legionella pneumophila*, did not observe in a transmission electron microscopy (TEM) analysis any bacterial cellular residues after only 2 h of photocatalytic treatment. The authors noted that the cellular organic substances have most likely been mineralized. Additionally, for a proven cell mineralization and quantitative analysis of the level of mineralization, the total organic carbon analysis was performed. After 75 min of photocatalytic oxidation, all total organic carbon (TOC) was removed, which indicated that the organic matter from bacterial cells can be totally oxidized by photocatalysis.

An almost complete degradation of *E. coli* bacterial cells after an approx. 4 h treatment on an activated carbon-supported TiO_2_ was confirmed by Youji et al. [54]. Bacterial cell decomposition was evaluated based on SEM photographs of bacteria on TiO_2_/AC before and after the photocatalytic process.

A thorough analysis of the literature has shown that in most cases, the presented results were only an initial stage of the bacterial cell photocatalytic mineralization process [55,56,57]. This is due, among other things, to the relatively short time of the experiments—less than 6 h (Table 2). The authors themselves also emphasized that they only studied the initial stages of the possible mineralization process. Rokicka et al. [57], for example, studied an initial stage of the photocatalytic mineralization of *E. coli* and *S. epidermidis* bacteria in the presence of carbon-modified TiO_2_ activated by UV-A and artificial solar light. Research has already shown that the 3 h photocatalytic process led to CO_2_ evolution from reaction suspension-contained bacteria. The amount of evolved CO_2_ increased gradually during the photocatalytic process. This might indicate that the initial stage of the photocatalytic bacterial mineralization process has been observed. The amount of evolved CO_2_ was relatively small, which might indicate that only the simple sugars, such as pentoses, hexoses, heptoses or amino sugars, and uronic acids composing the outer membrane (O-antigen in Gram-negative bacteria) or cell wall (Gram-positive bacteria), were decomposed.

An interesting approach to the examination of photocatalytic bacterial destruction was presented by Shi and co-workers [58]. The authors examined the inactivation of *E. coli* in the presence of Ag/AgX-CNTs (X = Cl, Br, I) plasmonic photocatalysts. In the presented work, a thorough analysis of the surface elemental composition of the photocatalysts, before and after photocatalytic treatment (in suspension containing *E. coli*) by an FT-IR analysis, was performed. It was observed that after the photocatalytic process, some additional absorption peaks on the surface of photocatalysts are observed. The observed bands can be attributed, among others, to the vibration of the sugar rings of the lipopolysaccharide (1096 cm^−1^), the –COO−group of the fatty acid (1395 cm^−1^), the glutamate carboxylate stretching (1577 cm^−1^), and oligosaccharide (1087 cm^−1^) and thus bands that can be attributed to bacterial cell residues. Admittedly, the authors did not directly use the mineralization term, but based on the obtained results, they noted that bacterial cells could be destructed to large biomolecules.

## 4. The Proposed Mechanism of Photocatalytic Bacterial Mineralization

One of the first possible mechanisms of photocatalytic bacterial mineralization was proposed by Sun et al. [51]. The authors suggested that *E. coli* photomineralization proceeds with a combination of UV photolysis breakdown upon mass cell and twofold mechanism, which involved the surface-controlled O_2_ adsorption and fission of organic cells on electron-rich and positive vacant sites. Inactivated bacterial cells have been successively attacked by highly reactive peroxy or hydroxyl radicals, which led to progressive destruction and finally mineralization to CO_2_ and H_2_O.

**Figure 2:**
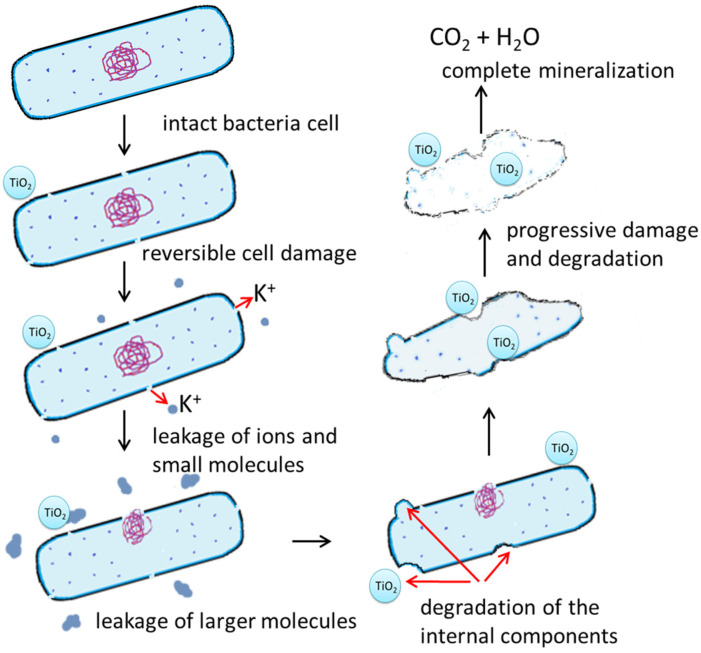
Scheme for proposed photocatalytic destruction and mineralization of bacteria in the presence of TiO_2_.

It is well known that in the first stage of bacterial destruction, bacterial cell inactivation must occurred. All presented in Section 2 mechanisms work together, ultimately leading to bacterial cell death. It is worth mentioning that already at this stage, the bacterial cell may present clear and significant damages (shape-shifting, membrane depressions, disruption of the cell membrane). Bacterial inactivation via membrane and cell wall damages, cytoplasmic membrane, or the leakage of intercellular components has been, for example, observed [24]. Research has shown that bacterial cell destruction could be initiated at any point on the cells. However, a statistical analysis showed that the bacterial cell damages most frequently appear on poles of rod-shaped *E. coli* cells [61,62]. The prolonged generation of free radicals leads to further and higher damage in bacterial cells. Membrane damage caused the leakage first of small molecules (such as ions) and further higher molecules, such as protein. Additionally, the protrusion of the cytoplasmic membrane and cytoplasmic components from degraded areas of the cell wall may have occurred. Simultaneous peroxidation processes of membrane wall components (e.g., lipopolysaccharide, phosphatidylethanolcholine, and peptidoglycan) are taking place. First comes the oxidation and mineralization of relatively small molecular compounds, such as simple sugars (e.g., pentoses, hexose forming part of lipo-polysaccharide) and uronic acids composing the outer membrane [57]. Next, phosphatidylethanolcholine and finally peptidoglycan are oxidized and mineralized [47]. If the photocatalytic process takes place in a suspension containing a suspended photocatalyst, the organic residual of decomposed bacteria, or even a part of a destructed cell, it may be absorbed on the surface of nanomaterials and be destructed to large biomolecules [58]. Bacterial cell could also be degraded to MDA (via the lipid peroxidation process), which in turn can be degraded to ring cleavage products (such as monoaldehydes, monoketones, carboxylic acids) and finally mineralized to CO_2_ and H_2_O. A simple scheme for the degradation and mineralization mechanism of TiO_2_ on bacteria is presented in Figure 2.

Kozlova et al. [22] observed that the mineralization of *B. thuringiensis* deposited on the surface of TiO_2_-covered glass slides proceeded in two stages. The division into stages was made on the basis of the rate of CO_2_ evolution. First comes the transformation part of bacteria and the large molecules of organic compounds constituting bacteria into the products of partial oxidation. This stage last approx. 30 h and is characterized by a gradual slow evolution of CO_2_. In the next stage, the products of partial oxidation are transformed into carbon dioxide; therefore, the rate of CO_2_ evolution increases. This stage can last up to 150 h.

## 5. Conclusions and Future Perspectives

According to the literature, TiO_2_ could photomineralize the bacterial cell, leaving no organic materials in purified water and on the surface of air purification filters. The results obtained so far indicate that the process of photocatalytic bacterial mineralization most probably proceeds in two stages. In the first stage, gradually, cell wall and membrane oxidation occurs. In the next stage, it comes to the mineralization of internal components that have been released from the injured cell. Unfortunately, due to large discrepancies in the timing of the various stages in studies conducted by different teams and researchers, it is difficult to determine the duration of the various stages of mineralization.

In reviewing the literature concerning photocatalytic microorganism mineralization, it was observed that this topic has not been thoroughly investigated. Therefore, an important aspect is further research on photocatalytic bacterial mineralization.

A significant aspect to be addressed in the future is the lack of standardization of conducted research in the field of photocatalytic microorganism inactivation and mineralization. The standardization of conducted studies will facilitate a comparison of future results. Additionally, the range of tested microorganisms should be expanded (such as fungi or algae).

Interestingly enough, as early as 2003, Sun et al. [51] mentioned that photocatalytic bacterial mineralization could improve the microbiology safety level of treated water by reducing the microorganism regrowth and elimination of biomass from the membrane. Gupta and Modak [63], in their critical review about the mechanistic aspects of photocatalysis microbial disinfection, noted that one of the aspects that reduces the activity of the photocatalyst and its reuse in subsequent cycles can be a deposition of dead bacterial cell mass on its surface. Considering that the deposition of a dead bacterial cell mass on the TiO_2_ surface can cause a decrease in photocatalytic activity, the examination of the bacterial cell mineralization and determination of optimal process parameters is useful from an economic point of view.

It is also worth expanding the range of research methods used to examine photocatalytic bacterial mineralization. Molecular techniques based on the quantification of DNA could be, for example, considered. These methods would allow for the detection of individual DNA or protein fragments after the photocatalytic process. It is also important to investigate the toxicity of the bacterial degradation intermediates during the photocatalytic mineralization process, e.g., by using methods based on bioluminescence technology to monitor contaminations.

## Figures and Tables

**Table 1:** Research conducted on photocatalytic microorganism destruction/mineralization in air.

Research Methods	Photocatalyst	Radiation Type	Used Microorganisms	Experiment Time	Results/Observations	Ref.
GC—CO_2_ measurements, ^14^C radioisotope labelling SEM analysis	P25 coated on glass	UV	*E. coli*	75 h	mineralization of bacteria	[18]
GC—CO_2_ measurements + kinetic data and carbon mass balance	P25 coated on quartz disks	UV	*E. coli*, *M. luteus*, *B. cereus*, *A. niger*	72–144 h	mineralization of microorganisms	[46]
FTIR spectrometer fitted with a gas cell—CO_2_ measurements, SEM analysis	metal microfibrous mesh coated with TiO_2_ (P25)	UV	*E. coli*	168 h	total remove bacteria from mesh	[49]
ATR-FTIR spectroscopy	P25 porous film	UV-A	*E. coli*	44 h	changes in spectral bands suggesting the mineralization	[47]
ATR-FTIR spectroscopy	P25 porous film	UV-A	*E. coli*	16 h	total oxidation of cell organic matter	[48]
GC—CO_2_ measurements	TiO_2_ (Hombifine N) and Pt/TiO_2_ on glass plates	UV	*B. thuringiensis*	50–150 h	full photocatalytic bacterial mineralization	[22]

GC—gas chromatography, PM—photocatalytic mineralization.

**Table 2:** Research conducted on photocatalytic microorganism destruction/mineralization in aqueous solutions.

Research Methods	Photocatalyst	Radiation Type	Used Microorganisms	Experiment Time	Results/Observations	Ref.
MDA formation GC—CO_2_ measurements	TiO_2_ or Ag-TiO_2_	UV	*E. coli*	40 min	complete mineralization of *E. coli*	[50]
GC—CO_2_ measurements, SEM analysis	TiO_2_–Fe_2_O_3_ powder	UV	*E. coli*	75 h	*E. coli* mineralization	[51]
TEM analysis, TOC analysis	P25	UV	*L. pneumophila*	2 h	bacterial mineralization	[53]
SEM analysis	TiO_2_/AC composites	UV	*E. coli*	250 min	nearly total bacterial decomposition	[54]
FT-IR analysis	Ag/AgX-CNTs (X = Cl, Br, I)	VL	*E. coli*	2 h	destruction of *E. coli* to large biomolecules	[58]
GC—CO_2_ measurements	Ag@TiO_2_	UV	*E. coli*	6 h	initial stage of bacterial mineralization	[55]
TOC analysis	Ag/TiO_2_-N	VL	*A. baumannii*	0.5 h	initial stage of bacterial mineralization	[59]
GC—CO_2_ measurements, SEM analysis	g-C_3_N_4_-SnO_2_/TiO_2_ nanotubes/Ti plates	VL	*E. coli*	32 h	complete mineralization of *E. coli*	[52]
FID-GC—CO_2_ measurements	Ag/TiO_2_	VL	*E. coli*	3 h	initial stage of bacterial mineralization	[56]
GC—CO_2_ measurements	TiO_2_ modified by carbon	UVA ASL	*E. coli* *S. epidermidis*	3 h	initial stage of bacterial mineralization	[57]
GC—CO_2_ measurements	TiO_2_/Ag_2_O/AuO NTs	VL	*S. aureus*	4 h	initial stage of bacterial mineralization	[60]

GC—gas chromatography, VL—visible light, ASL—artificial solar light, FID—GC—flame ionization detector for gas chromatography.

## Data Availability

The data presented in this study are available upon request from the corresponding author.

## References

[1] Liu H., Wang C., Wang G. (2020). Photocatalytic advanced oxidation processes for water treatment: Recent advances and perspective. Chem. Asian J..

[2] Verma V., Al-Dossari M., Singh J., Rawat M., Kordy M.G.M., Shaban M. (2022). A Review on green synthesis of TiO_2_ NPs: Photocatalysis and antimicrobial applications. Polymers.

[3] Eidsvåg H., Bentouba S., Vajeeston P., Yohi S., Velauthapillai D. (2021). TiO_2_ as a photocatalyst for water splitting—An experimental and theoretical review. Molecules.

[4] Li Z., Wang S., Wu J., Zhou W. (2022). Recent progress in defective TiO_2_ photocatalysts for energy and environmental applications. Renew. Sustain. Energy Rev..

[5] Banerjee S., Dionysiou D.D., Pillai S.C. (2015). Self-cleaning applications of TiO_2_ by photo-induced hydrophilicity and photocatalysis. Appl. Catal. B Environ..

[6] Al Jitan S., Palmisano G., Garlisi C. (2020). Synthesis and surface modification of TiO_2_-based photocatalysts for the conversion of CO_2_. Catalysts.

[7] Rafique M., Hajra S., Irshad M., Usman M., Imran M., Assiri M.A., Ashraf W.M. (2023). Hydrogen production using TiO_2_-based photocatalysts: A comprehensive review. ACS Omega.

[8] Chen D., Cheng Y., Zhou N., Chen P., Wang Y., Li K., Huo S., Cheng P., Peng P., Zhang R. (2020). Photocatalytic degradation of organic pollutants using TiO_2_-based photocatalysts: A review. J. Clean. Prod..

[9] Zeshan M., Bhatti I.A., Mohsin M., Iqbal M., Amjed N., Nisar J., Al Masoud L., Alomar T.S. (2022). Remediation of pesticides using TiO_2_ based photocatalytic strategies: A review. Chemosphere.

[10] Zhang B., Sun B., Liu F., Gao T., Zhou G. (2024). TiO_2_-based S-scheme photocatalysts for solar energy conversion and environmental remediation. Sci. China Mater..

[11] Jafari S., Mahyad B., Hashemzadeh H., Janfaza S., Gholikhani T., Tayebi L. (2020). Biomedical applications of TiO_2_ nanostructures: Recent advances. Int. J. Nanomed..

[12] Kodithuwakku P., Jayasundara D.R., Munaweera I., Jayasinghe R., Thoradeniya T., Weerasekera M., Ayajan P.A., Kottegoda N. (2022). A review on recent developments in structural modification of TiO_2_ for food packaging applications. Prog. Solid State Chem..

[13] Reddy P.V.L., Kavitha B., Reddy P.A.K., Kim K.H. (2017). TiO_2_-based photocatalytic disinfection of microbes in aqueous media: A review. Environ. Res..

[14] Demirel C.S.U., Birben N.C., Bekbolet M. (2018). A comprehensive review on the use of second generation TiO_2_ photocatalysts: Microorganism inactivation. Chemosphere.

[15] Amiri M.R., Alavi M., Taran M., Kahrizi D. (2022). Antibacterial, antifungal, antiviral, and photocatalytic activities of TiO_2_ nanoparticles, nanocomposites, and bio-nanocomposites: Recent advances and challenges. J. Public Health Res..

[16] Bono N., Ponti F., Punta C., Candiani G. (2021). Effect of UV Irradiation and TiO_2_-photocatalysis on airborne bacteria and viruses: An overview. Materials.

[17] De Pasquale I., Porto C.L., Dell’Edera M., Curri M.L., Comparelli R. (2021). TiO_2_-based nanomaterials assisted photocatalytic treatment for virus inactivation: Perspectives and applications. Curr. Opin. Chem. Eng..

[18] Jacoby W.A., Maness P.C., Wolfrum E.J., Blake D.M., Fennell J.A. (1998). Mineralization of bacterial cell mass on a photocatalytic surface in air. Environ. Sci. Technol..

[19] Mudhoo A., Paliya S., Goswami P., Singh M., Lofrano G., Carotenuto M., Libralato G., Guida M., Usman M., Kumar S. (2020). Fabrication, functionalization and performance of doped photocatalysts for dye degradation and mineralization: A review. Environ. Chem. Lett..

[20] Adeyemi J.O., Ajiboye T., Onwudiwe D.C. (2021). Mineralization of antibiotics in wastewater via photocatalysis. Water Air Soil Pollut..

[21] Kumar S., Sharma R., Gupta A., Dubey K.K., Khan A.M., Singhal R., Kumar R., Bharti A., Singh P., Kant R. (2022). TiO_2_ based Photocatalysis membranes: An efficient strategy for pharmaceutical mineralization. Sci. Total Environ..

[22] Kozlova E.A., Safatov A.S., Kiselev S.A., Marchenko V.Y., Sergeev A.A., Skarnovich M.O., Emelyanova E.K., Smetannikova M.A., Buryak G.A., Vorontsov A.V. (2010). Inactivation and mineralization of aerosol deposited model pathogenic microorganisms over TiO_2_ and Pt/TiO_2_. Environ. Sci. Technol..

[23] Taddese R., Belzer C., Aalvink S., de Jonge M.I., Nagtegaal I.D., Dutilh B.E., Boleij A. (2021). Production of inactivated gram-positive and gram-negative species with preserved cellular morphology and integrity. J. Microbiol. Methods.

[24] Foster H.A., Ditta I.B., Varghese S., Steele A. (2011). Photocatalytic disinfection using titanium dioxide: Spectrum and mechanism of antimicrobial activity. Appl. Microbiol. Biotechnol..

[25] Matsunaga T., Tomoda R., Nakajima T., Wake H. (1985). Photochemical sterilization of microbial cells by semiconductor powder. FEMS Microbiol. Lett..

[26] Dalrymple O.K., Stefanakos E., Trotz M.A., Goswami D.Y. (2010). A review of the mechanisms and modeling of photocatalytic disinfection. Appl. Catal. B Environ..

[27] Pagnout C., Jomini S., Dadhwal M., Caillet C., Thomas F., Bauda P. (2012). Role of electrostatic interactions in the toxicity of titanium dioxide nanoparticles toward *Escherichia coli*. Colloids Surf. B.

[28] Liao D.L., Wu G.S., Liao B.Q. (2009). Zeta potential of shape-controlled TiO_2_ nanoparticles with surfactants. Colloids Surf. A Physicochem. Eng. Asp..

[29] Shah A.H., Rather M.A. (2021). Effect of calcination temperature on the crystallite size, particle size and zeta potential of TiO_2_ nanoparticles synthesized via polyol-mediated method. Mater. Today Proc..

[30] Saito T., Iwase T., Horie J., Morioka T. (1992). Mode of photocatalytic bactericidal action of powdered semiconductor TiO_2_ on mutans streptococci. J. Photochem. Photobiol. B Biol..

[31] Hu C., Guo J., Qu J., Hu X. (2007). Photocatalytic degradation of pathogenic bacteria with AgI/TiO_2_ under visible light irradiation. Langmuir.

[32] Gogniat G., Thyssen M., Denis M., Pulgarin C., Dukan S. (2006). The bactericidal effect of TiO_2_ photocatalysis involves adsorption onto catalyst and the loss of membrane integrity. FEMS Microbiol. Lett..

[33] Maness P.C., Smolinski S., Blake D.M., Huang Z., Wolfrum E.J., Jacoby W.A. (1999). Bactericidal activity of photocatalytic TiO_2_ reaction: Toward an understanding of its killing mechanism. Appl. Environ. Microbiol..

[34] Kiwi J., Nadtochenko V. (2004). New evidence for TiO_2_ photocatalysis during bilayer lipid peroxidation. J. Phys. Chem. B.

[35] Carré G., Hamon E., Ennahar S., Estner M., Lett M.C., Horvatovich P., Gies J.P., Andre P. (2014). TiO_2_ photocatalysis damages lipids and proteins in *Escherichia coli*. Appl. Environ. Microbiol..

[36] Koizumi Y., Yamada R., Nishioka M., Matsumura Y., Tsuchido T., Taya M. (2002). Deactivation kinetics of *Escherichia coli* cells correlated with intracellular superoxide dismutase activity in photoreaction with titanium dioxide particles. J. Chem. Technol. Biotechnol..

[37] Kumar A., Pandey A.K., Singh S.S., Shanker R., Dhawan A. (2011). Engineered ZnO and TiO_2_ nanoparticles induce oxidative stress and DNA damage leading to reduced viability of *Escherichia coli*. Free. Radic. Biol. Med..

[38] Rokicka-Konieczna P., Wanag A., Sienkiewicz A., Kusiak-Nejman E., Morawski A.W. (2021). Effect of APTES modified TiO_2_ on antioxidant enzymes activity secreted by *Escherichia coli* and *Staphylococcus epidermidis*. Biochem. Biophys. Res. Commun..

[39] Zhang H.M., Cao J., Tang B.P., Wang T.G. (2014). Effect of TiO_2_ nanoparticles on the structure and activity of catalase. Chem. Biol. Interact..

[40] Gogniat G., Dukan S. (2007). TiO_2_ photocatalysis causes DNA damage via Fenton reaction-generated hydroxyl radicals during the recovery period. Appl. Environ. Microbiol..

[41] Liu Y., Huang J., Feng X., Li H. (2021). Thermal-sprayed photocatalytic coatings for biocidal applications: A review. J. Therm. Spray Technol..

[42] Vohra A., Goswami D.Y., Deshpande D.A., Block S.S. (2006). Enhanced photocatalytic disinfection of indoor air. Appl. Catal. B Environ..

[43] Rao N.G., Kumar A., Wong J.S., Shridhar R., Goswami D.Y. (2018). Effect of a novel photoelectrochemical oxidation air purifier on nasal and ocular allergy symptoms. Ther. Adv. Allergy Rhinol..

[44] Goswami D.Y. (1997). A review of engineering developments of aqueous phase solar photocatalytic detoxification and disinfection processes. J. Sol. Energy Eng..

[45] Goswami T.K., Hingorani S.K., Greist H., Goswami D.Y., Block S.S. (1999). Photocatalytic system to destroy bioaerosols in air. J. Adv. Oxid. Technol..

[46] Wolfrum E.J., Huang J., Blake D.M., Maness P.C., Huang Z., Fiest J., Jacoby W.A. (2002). Photocatalytic oxidation of bacteria, bacterial and fungal spores, and model biofilm components to carbon dioxide on titanium dioxide-coated surfaces. Environ. Sci. Technol..

[47] Kiwi J., Nadtochenko V. (2005). Evidence for the mechanism of photocatalytic degradation of the bacterial wall membrane at the TiO_2_ interface by ATR-FTIR and laser kinetic spectroscopy. Langmuir.

[48] Nadtochenko V.A., Sarkisov O.M., Nikandrov V.V., Chubukov P.A., Denisov N.N. (2008). Inactivation of pathogenic microorganisms in the photocatalytic process on nanosized TiO_2_ crystals. Russ. J. Phys. Chem. B.

[49] López J.E.O., Jacoby W.A. (2002). Microfibrous mesh coated with titanium dioxide: A self-sterilizing, self-cleaning filter. J. Air Waste Manag. Assoc..

[50] Sökmen M., Candan F., Sümer Z. (2001). Disinfection of *E. coli* by the Ag-TiO_2_/UV system: Lipidperoxidation. J. Photochem. Photobiol. A Chem..

[51] Sun D.D., Tay J.H., Tan K.M. (2003). Photocatalytic degradation of *E. coliform* in water. Water Res..

[52] Faraji M., Mohaghegh N., Abedini A. (2018). Ternary composite of TiO_2_ nanotubes/Ti plates modified by g-C_3_N_4_ and SnO_2_ with enhanced photocatalytic activity for enhancing antibacterial and photocatalytic activity. J. Photochem. Photobiol. B.

[53] Cheng Y.W., Chan R.C., Wong P.K. (2007). Disinfection of *Legionella pneumophila* by photocatalytic oxidation. Water Res..

[54] Youji L.I., Mingyuan M.A., Xiaohu W.A.N.G., Xiaohua W.A.N.G. (2008). Inactivated properties of activated carbon-supported TiO_2_ nanoparticles for bacteria and kinetic study. J. Environ. Sci..

[55] Kowalska E., Wei Z., Karabiyik B., Herissan A., Janczarek M., Endo M., Markowska-Szczupak A., Remita H., Ohtani B. (2015). Silver-modified titania with enhanced photocatalytic and antimicrobial properties under UV and visible light irradiation. Catal. Today.

[56] Endo M., Wei Z., Wang K., Karabiyik B., Yoshiiri K., Rokicka P., Ohtani B., Markowska-Szczupak A., Kowalska E. (2018). Noble metal-modified titania with visible-light activity for the decomposition of microorganisms. Beilstein J. Nanotechnol..

[57] Rokicka-Konieczna P., Markowska-Szczupak A., Kusiak-Nejman E., Morawski A.W. (2019). Photocatalytic water disinfection under the artificial solar light by fructose-modified TiO_2_. Chem. Eng. J..

[58] Shi H., Li G., Sun H., An T., Zhao H., Wong P.K. (2014). Visible-light-driven photocatalytic inactivation of *E. coli* by Ag/AgX-CNTs (X = Cl, Br, I) plasmonic photocatalysts: Bacterial performance and deactivation mechanism. Appl. Catal. B Environ..

[59] Yang G., Yin H., Liu W., Yang Y., Zou Q., Luo L., Li H., Huo Y., Li H. (2018). Synergistic Ag/TiO_2_-N photocatalytic system and its enhanced antibacterial activity towards *Acinetobacter baumannii*. Appl. Catal. B Environ..

[60] Kozak M., Mazierski P., Żebrowska J., Klimczuk T., Lisowski W., Żak A.M., Skowron P.M., Zaleska-Medynska A. (2024). Detailed insight into photocatalytic inactivation of pathogenic bacteria in the presence of visible-light-active multicomponent photocatalysts. Nanomaterials.

[61] Liou J.W., Chang H.H. (2012). Bactericidal effects and mechanisms of visible light-responsive titanium dioxide photocatalysts on pathogenic bacteria. Arch. Immunol. Ther. Exp..

[62] Liou J.W., Gu M.H., Chen Y.K., Chen W.Y., Chen Y.C., Tseng Y.H., Hung J.L., Chang H.H. (2011). Visible light responsive photocatalyst induces progressive and apical-terminus preferential damages on *Escherichia coli* surfaces. PLoS ONE.

[63] Gupta R., Modak J. (2020). Bacterial lysis via photocatalysis-A critical mechanistic review. ChemCatChem.

